# Assessment of Turnaround Time (TAT) for Oral Squamous Cell Carcinoma Biopsies: A Single-Institution Experience

**DOI:** 10.7759/cureus.62677

**Published:** 2024-06-19

**Authors:** Priyadharshini G, Karthikeyan Ramalingam, Pratibha Ramani, Deepak Nallaswamy

**Affiliations:** 1 Oral Pathology and Microbiology, Saveetha Dental College and Hospitals, Saveetha Institute of Medical and Technical Sciences, Saveetha University, Chennai, IND; 2 Prosthodontics, Saveetha Dental College and Hospitals, Saveetha Institute of Medical and Technical Sciences, Saveetha University, Chennai, IND

**Keywords:** reporting, quality improvement study, laboratory administration, oral pathology, excisional biopsy, biopsy, incisional biopsy, oral squamous cell carcinoma, patient outcome, turnaround time

## Abstract

Background: Oral squamous cell carcinoma (OSCC) is one of the most common cancers worldwide. A delay in the diagnosis of OSCC can have a drastic impact on management and patient outcomes. One of the most crucial elements in oral management is the timely histopathological final diagnosis. Turnaround time (TAT) is regarded as the most important component of the quality performance evaluation. Many labs have struggled to improve their TATs despite advancements in computerization, transport systems, and analytical technologies.

Aim: This study aimed to assess the TAT of OSCC cases, assess the mean TAT period, evaluate any TAT delays, and explore the reasons behind the TAT delays.

Materials and methods: OSCC reports in Saveetha Dental College and Hospitals, Chennai, for one year from January 1, 2022, to December 31, 2022, were retrieved from the Dental Information Archival Software (DIAS), and the mean TAT was noted. Further, the number of cases with delay in TAT was also observed, and the reason for their delay was listed. Descriptive statistics and graphical representation were performed utilizing IBM SPSS Statistics for Windows, V. 23.0 (IBM Corp., Armonk, NY, USA). One-way ANOVA was performed with a significance set at a p-value less than 0.05.

Results: 230 OSCC cases were retrieved and included in the TAT evaluation for this study. Among 230 cases, 161 (70%) were incisional and 69 (30%) were excisional biopsies. Only seven (4%) incisional cases and seven (10%) excisional biopsies showed a delay in TAT. The most common reason for the delay in TAT was the requirement for deeper sections and decalcification of bone specimens. Out of 161 incisional cases, only 48 (29%) have undergone excision and further treatment. Twenty-one out of 69 (30%) excisional cases were found to be referral cases from other private institutions. The overall average TAT for 12 months was 3.24 ± 0.41 days for incisional biopsies and 11.88 ± 2.07 days for excisional biopsies. One-way ANOVA revealed a statistically significant p-value of less than 0.00001.

Conclusion: Our study sheds light on specific challenges in TAT delay and opportunities for the improvement of TAT. This can result in faster TAT of OSCC reports, further improve patient care, and enable prompt treatment. This study quantified the TAT for OSCC cases and identified critical areas for process improvement. The findings can inform strategies to streamline diagnostic workflows, reduce delays, and ultimately improve the timely delivery of care to patients with OSCC.

## Introduction

Oral squamous cell carcinoma (OSCC) is one of the most common cancers worldwide and is linked to high rates of both mortality and morbidity, making it a global public health concern [1]. With the current chemo-radiotherapeutic procedures, even slight delays in applying additional interventions may affect patient outcomes. It may lead to increased tumor invasion and infiltration of the loco-regional tissue, a higher risk of metastasis, and chances that a previously resectable growth could become unmanageable or unresectable [2]. One of the most crucial elements in oral cancer management is the timely histopathological final diagnosis [3]. 

Every choice of cancer care is impacted by its pathology report including the diagnosis, cancer subtype, stage, and relevant prognostic data. It includes whether or not a patient has a treatable illness and whether or not it influences the course of treatment [4]. Patients and their families who are waiting for a diagnosis and treatment plan also experience more stress as a result, particularly when their medical practitioners are unable to estimate when the results will be available [5]. Clinical reports of disease progress show the scope for the promising application of mRNA-based research and therapeutics [6-8].

The period between the lab's accession of the material and the release of the final pathology report is referred to as turnaround time (TAT). Timeliness is possibly the most crucial of these attributes to the physician, who could be willing to forgo analytical quality in favor of a quicker TAT [9]. It is one of the important measures of laboratory performance and is a great measure that partially captures laboratory quality [10]. TAT is regarded as the most important component of the daily quality performance evaluation for several reasons: it is easy to measure using laboratory information systems; it has a significant financial impact on cost-effectiveness; and it is included in the calculation of physician satisfaction indicators [9].

In surgical pathology, prompt completion of reports and accurate diagnosis are key performance metrics [11]. A timely report that is sent can assist a doctor in making a diagnosis and starting a treatment plan, while a report that is delayed needlessly can jeopardize the patient's care [12]. Studies reveal that longer TATs increase mortality by 1.8% per day delay in the diagnosis of early-stage cancer [13]. Reports that are issued slowly lead to longer patient treatment wait times, worse patient satisfaction, and higher hospital expenses [14].

A significant problem in understanding between the surgeon and the pathologist is due to unsatisfactory TAT, which takes a lot of time and effort for the staff to resolve and improve. Many labs have struggled to improve their TATs despite advancements in computerization, transport systems, and analytical technologies [15].

This study aimed to evaluate the TAT of cases involving OSCC, analyze the average TAT, investigate any delays in TAT, and identify the factors contributing to these TAT delays.

## Materials and methods

This retrospective study was conducted after obtaining ethical clearance from the Institutional Human Ethical Committee of Saveetha Dental College and Hospitals, Saveetha Institute of Medical and Technical Sciences, Chennai, India (vide reference number: IHEC/SDC/PhD/OPath-1954/19/TH-001). It was obtained to ensure compliance with institutional, national, and international ethical guidelines.

This study was conducted at the Department of Oral Pathology, Saveetha Dental College and Hospitals. It was focused on cases of OSCC diagnosed through incisional and excisional biopsies. The selection spanned a year from January 1, 2022, to December 31, 2022. The primary objective was to evaluate the TAT for histopathological reporting, defined as the duration from specimen arrival at the lab to the delivery of the final histopathological report. Data for this study were meticulously gathered using the institution's Dental Information Archival Software (DIAS), ensuring precise and comprehensive information retrieval.

The study distinguished between cases reported within the expected timeframe of four working days for incisional biopsy and 14 working days for excisional biopsy and those with delayed reporting beyond the TAT deadline. The average TAT was calculated separately for incisional and excisional biopsies. Additionally, the mean number of TAT delays was calculated. We tried to identify and explore common reasons for delays that can provide insights into potential bottlenecks in the diagnostic process.

The study also tracked the number of cases initially diagnosed through incisional biopsies that subsequently underwent excisional biopsies. This data was crucial for understanding the diagnostic and treatment protocol for OSCC. A subset of cases referred from other private institutions were also identified. We also assessed the referral patterns and whether there were differences in TAT between in-house and referred cases. All collected data were organized and tabulated in Microsoft Excel. This step ensured that the data was readily accessible and analyzable.

Descriptive statistics were employed to summarize the data. Mean values for various metrics were calculated to understand the general trends in TAT. IBM SPSS Statistics for Windows, V. 23.0 (IBM Corp., Armonk, NY, USA) was used for statistical analysis, enabling precise calculations and facilitating the creation of graphical representations of the data. One-way ANOVA was performed with a statistical significance set at a p-value of less than 0.05.

## Results

**Figure 1:**
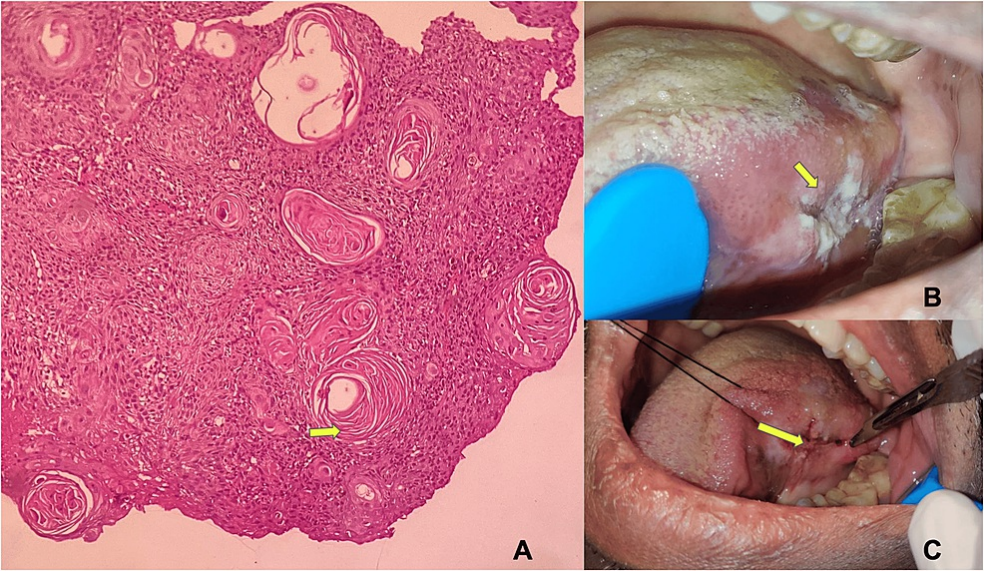
Incisional biopsy images (A) Photomicrograph showing malignant epithelial islands with keratin pearl production (H&E, 10×). (B) Clinical picture showing the ulcerated, endophytic tumor involving the lateral border of the tongue. (C) Intraoperative picture showing the incisional biopsy procedure. H&E: hematoxylin and eosin

**Figure 2:**
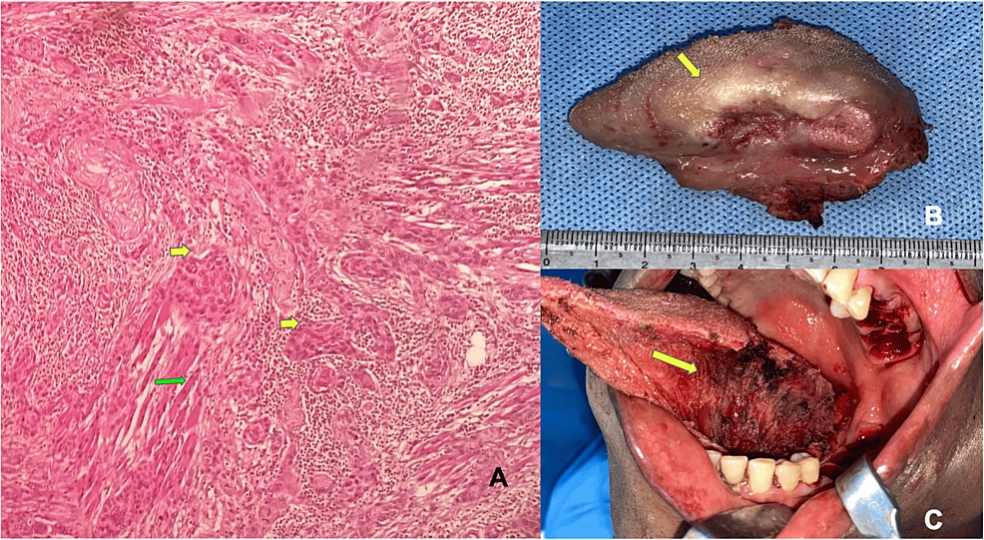
Excisional biopsy images (A) Photomicrograph showing malignant epithelial islands (yellow arrow) with invasion into skeletal muscle (green arrow) (H&E, 10×). (B) Image of the excised specimen. (C) Postoperative picture after surgical excision. H&E: hematoxylin and eosin

Eight hundred ninety-four total biopsies were received in our department in 2022. Two hundred thirty (25.72%) cases were diagnosed as OSCC. Out of 230 included OSCC cases, 161 (70%) were incisional biopsies (Figure 1), and 69 (30%) were excisional biopsies (Figure 2).

Figure 2 shows the excisional biopsy of the malignant tongue tumor and its histopathology revealing squamous cell carcinoma with invasion into skeletal muscles. 

The mean TAT for incisional OSCC cases varied throughout the year 2022 as follows: January, 3.35 days; February, 3.00 days; March, 3.07 days; April, 2.52 days; May, 2.70 days; June, 3.22 days; July, 3.15 days; August, 3.84 days; September, 3.24 days; October, 3.90 days; November, 3.05 days; and December, 3.70 days. The overall average TAT for incisional biopsies in 2022 was 3.245 days. The mean TAT for excisional OSCC cases in 2022 was as follows: January, 11.5 days; February, 11.75 days; March, 7.4 days; April, 11.0 days; May, 11.6 days; June, 11.8 days; July, 10.6 days; August, 13.12 days; September, 12.7 days; October, 11.25 days; November, 13.75 days; and December, 16.2 days. The overall average TAT for excisional biopsies was 11.88 days (Table 1).

**Table 1:** The mean TAT of all the incisional and excisional OSCC cases reported from January to December 2022 Data is presented as a monthly mean ± standard deviation of TAT (in days). TAT: turnaround time; OSCC: oral squamous cell carcinoma

Months recorded in 2022	Mean TAT ± standard deviation for incisional biopsies (days)	Mean TAT ± standard deviation for excisional biopsies (days)
January	3.55 ± 0.52	11.5 ± 1.29
February	3 ± 0.75	11.75 ± 1.5
March	3.07 ± 0.73	7.4 ± 4.97
April	2.52 ± 0.86	11 ± 1.27
May	2.7 ± 0.86	11.6 ± 2.13
June	3.22 ± 1.26	11.8 ± 2.38
July	3.15 ± 0.68	10.6 ± 1.5
August	3.84 ± 1.38	13.12 ± 3.31
September	3.24 ± 1.44	12.7 ± 2.49
October	3.9 ± 2.19	11.25 ± 3.09
November	3.05 ± 1.27	13.75 ± 2.07
December	3.7 ± 1.64	16.2 ± 4.6
Overall average for 12 months	3.24 ± 0.412	11.88 ± 2.07

**Figure 3:**
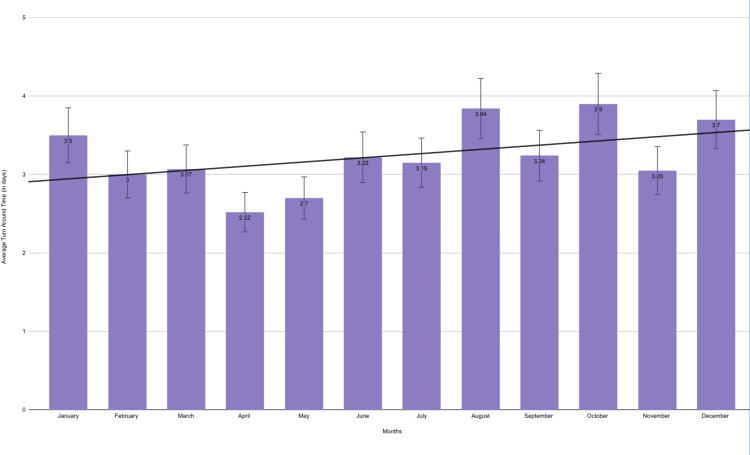
Graphical representation showing the TAT for the incisional OSCC cases for 12 months Data is presented as a monthly mean of TAT (in days). TAT: turnaround time; OSCC: oral squamous cell carcinoma

The mean TAT of incisional biopsies is shown in Figure 3. The lowest mean TAT of 2.52 days was observed in April, and the highest mean TAT of 3.9 days was observed in October.

**Figure 4:**
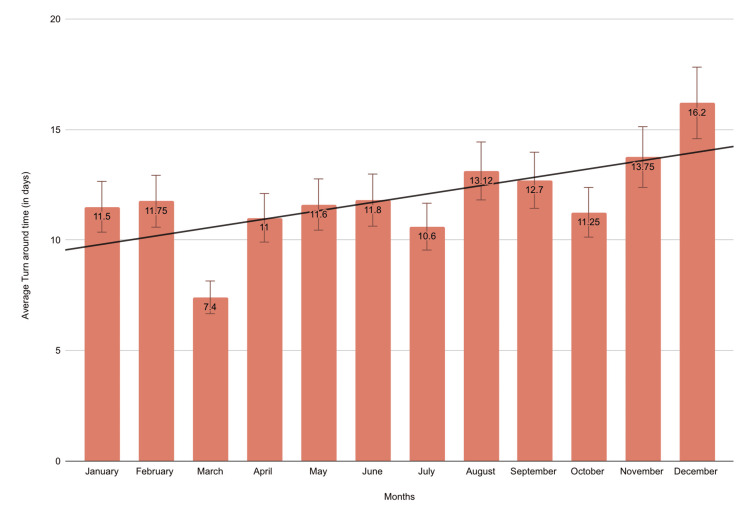
Graphical representation showing the TAT for the excisional OSCC cases for 12 months Data is presented as a monthly mean of TAT (in days). TAT: turnaround time; OSCC: oral squamous cell carcinoma

The mean TAT of excisional biopsies is shown in Figure 4. The lowest mean TAT of 7.4 days was observed in March, and the highest mean TAT of 16.2 days was seen in December.

The target TAT for reporting incisional cases at our institution was four days, while for excisional cases, it was 14 days. Of the 161 incisional cases, 154 (96%) were reported within the TAT, and only seven (4%) exceeded the TAT. Among the 69 excisional cases, 62 (90%) met the TAT, and seven (10%) were reported with a delay (Table 2).

**Table 2:** Table showing the TAT summary observed in our study TAT: turnaround time

Type of biopsy	Total number of cases (n)	Number of cases reported on TAT (n (%))	Number of cases reported with delay in TAT (n (%))
Incisional biopsy	161	154 (96%)	7 (4%)
Excisional biopsy	69	62 (90%)	7 (10%)

**Figure 5:**
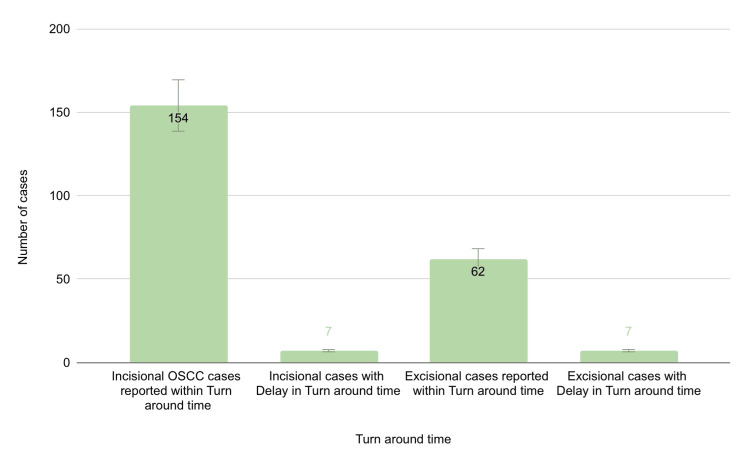
Graphical representation showing the number of cases reported on time and the number of cases with delay in TAT Bars represent the number of cases. TAT: turnaround time; OSCC: oral squamous cell carcinoma

On average, there was a delay of 7.4 days for reporting incisional OSCC cases beyond the TAT and 17.5 days for excisional OSCC cases. Of the seven incisional cases delayed beyond the TAT, the primary reason for the delay in four cases (57%) was the need for deeper tissue samples to assess invasion. Other reasons included the need for special stains (one case, 14%), immunohistochemistry (one case, 14%), and lack of clinical information (one case, 14%). Among the seven excisional cases with delayed reporting, six cases (85%) required longer decalcification times for larger maxillary or mandibular excised bone. Additionally, it was observed that out of 161 cases reported as OSCC in incisional biopsies, only 48 cases (29%) proceeded to excision and further treatment. Furthermore, 21 out of the 69 excisional cases (30%) were referral cases from other private institutions (Figure 5).

The monthly TAT of incisional and excisional biopsies was compared with one-way ANOVA. The mean TAT of incisional biopsy was 3.245 with a standard deviation of 0.4311. The mean TAT of excisional biopsy was 11.8892 with a standard deviation of 2.0779. The annual mean TAT was 7.567 with a standard deviation of 4.6526 (Table 3).

**Table 3:** Comparison between incisional and excisional biopsy monthly TAT TAT: turnaround time

	Mean TAT (in days)			
	Incisional biopsy (a)	Excisional biopsy (b)	Total (a + b)	
N	12	12	24	
∑X	38.94	142.67	181.61	
Mean	3.245	11.8892	7.567	
∑X^2^	128.4044	1743.7219	1872.1263	
Std. dev.	0.4311	2.0779	4.6526	

One-way ANOVA was performed for the mean TAT of our study samples. It was calculated between the groups and within the groups. The f-ratio value is 199.10242. The p-value was <0.00001. The result was significant at p <0.05 (Table 4).

**Table 4:** One-way ANOVA among the studied samples The f-ratio value is 199.10242. The p-value is <0.00001. The result is significant at p <0.05. a: mean TAT of incisional biopsies; b: mean TAT of excisional biopsies; DF: the degrees of freedom in the source; SS: the sum of squares due to the source; MS: the mean sum of squares due to the source. F: the F-statistic which is equal to the mean square of the between groups divided by the mean square of the within groups; TAT: turnaround time

Source	SS	df	MS	f-ratio
Between a and b	448.3297	1	448.3297	F = 199.10242
Within a and b	49.5386	22	2.2518	
Total	497.8683	23	-	

## Discussion

A clinician's suspicion of oral cancer drives the choice to perform a biopsy on a patient. Both surgeons and patients anticipate reliable and prompt surgical pathology results for treatment planning [16]. Short TATs in surgical pathology are further compelled by economic concerns like the need to shorten hospital stays and settle expenses quickly following discharge from the hospital [11]. TAT is one of the most obvious indicators of a laboratory service, and many physicians use it to assess the caliber of the laboratory. Since our study focused only on OSCC TAT, delays in reporting OSCC could have postponed treatment initiation, leading to disease progression and potentially poorer outcomes. Furthermore, uncertainty about one's health status due to delayed reporting can cause significant anxiety and distress for patients and their families, impacting their overall well-being [17].

Chan et al. [5] reported a mean TAT of 11.55 ± 11.38 days for oral histopathology specimens. An audit of surgical pathology by Malami and Iliyasu in Kano, Nigeria, found a TAT range of 2-16 days with a mean of 6.2 days, with nearly 75% of specimen reporting completed within seven days [18]. In comparison, our study found a mean TAT of 3.24 ± 0.412 days for incisional biopsies of OSCC and 11.88 ± 2.07 days for excisional biopsies of OSCC.

Studies suggest that longer TAT can be caused by larger specimen volumes, delayed slide delivery, and integration of pathology trainees [17]. The number of employees, automation level, and institution size are also a few variables that could impact the laboratory's mean TAT [18]. The total number of cases of TAT delay from our institution was less compared to other hospitals [18]. This may be attributed to our institution's digital initiative using online software called DIAS which enables easy tracking and monitoring of all the steps involved from biopsy procedures to histopathological reporting. 

We observed that 4% and 10% of the incisional and excisional cases, respectively, had TAT delay. The mean TAT in our study was found to be higher for both incisional and excisional biopsy compared to a previous study conducted in our institution by Georgia et al. [19] wherein the mean TAT was found to be three days and 7.4 days for incisional and excisional biopsy, respectively. They had compared the TAT of all biopsies received in the department while our study was focused only on OSCC biopsies. The increase in TAT in the present study could be due to a lack of prioritization of pending cases. It caused a delay in the reporting of a few cases, while others were processed more quickly.

Timely diagnosis and treatment are critical for oral cancer patients, and high priority is needed for OSCC reporting. The delay was found to be greater in excisional OSCC cases compared to incisional OSCC cases. Time taken for complex surgical specimens must be shortened by identifying the critical TAT components [3]. In our study, the most common cause for delay in excision specimens was the longer number of days taken for the decalcification of maxillectomy or mandibular complex specimens. Patel et al. reported that the most common reason for the delay in TAT was the need for immunohistochemistry analysis [3]. Jerjes et al. in their study found that the primary delay in TAT was 16 days and attributed it to the longer processing time for larger specimens [2].

Further, 30% of the excisional cases were found to be referred from other private centers. The specimens from other private centers usually lack relevant clinical details, and the time required for retrieving clinical information further delays the reporting. Ali et al. reported a shorter TAT with cases having concise and clear clinical pieces of information. Long and thorough clinical information is frequently observed when the surgical specimen is complicated and takes longer to report [12]. Thus, detailed communication between the surgeon and the pathologist is essential in improving TAT. 

A multifaceted approach is needed to address the underlying causes of delayed reporting of oral cancer cases [20]. This approach should include streamlining reporting procedures through technology, enhancing communication channels, ensuring adequate staffing levels, implementing quality improvement initiatives, and optimizing workflow processes. By prioritizing prompt reporting and early intervention, healthcare practitioners can improve treatment outcomes for individuals with oral cancer and reduce the associated challenges and consequences [21].

The limitation of our study is that variables like sample size, institutional variances, and potential biases may impact the generalizability. We have evaluated only the TAT of OSCC cases received in our department. TAT variations in other neoplasms and infectious diseases are underway in our institution. TAT variations in cytology requests, microbiology samples, and serological tests could be included in future analyses. Further multi-centric research should investigate the factors that might affect TAT and propose methods to limit them.

## Conclusions

Improving TAT in oral cancer reporting is essential as delayed TAT can impact patient outcomes and clinical decision-making. Our retrospective study on the evaluation of TAT in oral cancer cases has yielded important information on the average TAT and the reasons for delay in TAT. This assessment would provide information to develop ideas to improve TAT in oral cancer reporting. It is not merely an operational enhancement but a critical factor in delivering high-quality patient care. The insights gained from our retrospective study will provide a foundation for developing targeted strategies to reduce delays. By addressing the identified factors and implementing recommended improvements, we can achieve earlier diagnoses, prompt treatment, and ultimately better health outcomes for patients with oral cancer.
